# Prevalence and molecular detection of fluoroquinolone-resistant genes (*qnrA* and *qnrS*) in *Escherichia coli* isolated from healthy broiler chickens

**DOI:** 10.14202/vetworld.2018.1720-1724

**Published:** 2018-12-24

**Authors:** Shahin Mahmud, K. H. M. Nazmul Hussain Nazir, Md. Tanvir Rahman

**Affiliations:** Department of Microbiology and Hygiene, Faculty of Veterinary Science, Bangladesh Agricultural University, Mymensingh 2202, Bangladesh

**Keywords:** *Escherichia coli*, healthy broiler chickens, polymerase chain reaction, *qnrA*, *qnrS*, quinolone resistance

## Abstract

**Aim:**

The present study was carried out to determine the prevalence and molecular detection of fluoroquinolone-resistant *Escherichia coli* carrying *qnrA* and *qnrS* genes in healthy broiler chickens in Mymensingh, Bangladesh, and also to identify the genes responsible for such resistance.

**Materials and Methods:**

A total of 65 cloacal swabs were collected from apparently healthy chickens of 0-14 days (n=23) and 15-35 days (n=42) old. The samples were cultured onto Eosin Methylene Blue Agar, and the isolation and identification of the *E. coli* were performed based on morphology, cultural, staining, and biochemical properties followed by polymerase chain reaction (PCR) targeting *E. coli* 16S rRNA genes. The isolates were subjected to antimicrobial susceptibility test against five commonly used antibiotics under fluoroquinolone (quinolone) group, namely gatifloxacin, levofloxacin, moxifloxacin, ofloxacin, and pefloxacin by disk diffusion method. Detection of *qnrA* and *qnrS* genes was performed by PCR.

**Results:**

Among the 65 cloacal samples, 54 (83.08%) were found to be positive for *E. coli*. Antibiotic sensitivity test revealed that, of these 54 isolates, 18 (33.33%) were found to be resistant to at least one fluoroquinolone antibiotic. The highest resistance was observed against pefloxacin (61.11%). By PCR, of 18 *E. coli* resistant to fluoroquinolone, 13 (72.22%) were found to be positive for the presence of *qnrS*. None of the isolates were found positive for *qnrA*.

**Conclusion:**

Fluoroquinolone-resistant *E. coli* harboring *qnrS* genes is highly prevalent in apparently healthy broiler chickens and possesses a potential threat to human health.

## Introduction

Antibiotic resistance is an ever-increasing multinational public health crisis. It causes an estimated 700,000 deaths each year across the world [1]. Modern food animal production depends on the use of large amount of antibiotics for disease control and agricultural purposes, particularly for growth enhancement. This increased use of antibiotics is known to be a major factor for the development of antibiotic resistance in bacteria. Antibiotic-resistant bacterial species are widely distributed in both healthy and diseased animals and birds. Diseases caused by antibiotic-resistant bacteria are very difficult to treat. Poultry eggs and meats are major sources of human dietary ­protein. In Bangladesh poultry is raised in close association with human. From healthy birds, these antibiotic-resistant bacteria can spread directly to human or indirectly through contaminated egg and meat, for example, through the food chain to cause severe health problems [2]. *Escherichia coli* is a Gram-negative enteric bacterium of the family Enterobacteriaceae. They are one of the main species of bacteria that live in the lower intestines of warm-blooded animals and birds. While most of the *E. coli* strains are non-pathogenic, some of them can cause a variety of intestinal and extraintestinal infections in men, animals, and poultry [3]. In the poultry industry, colibacillosis, a disease caused by *E. coli*, significantly contributes to increased mortality and economic losses [4].

Fluoroquinolone (quinolones) antibiotics are a group of synthetic broad-spectrum bactericidal chemical agents having a bicyclic core structure related to 4-quinolone. They were introduced first in the form of nalidixic acid in the 1960s. This fully synthetic agent was discovered as a by-product of research on antimalarial drugs. Fluoroquinolones are also one of the most commonly used antibiotics, particularly in poultry. Fluoroquinolones interfere bacterial DNA synthesis by inhibiting topoisomerase class enzymes, namely DNA gyrase (topoisomerase II) in Gram-negative bacteria and topoisomerase IV in Gram-positive microorganisms [5]. Resistance against fluoroquinolone occured is primarily due to activities of products of genes such as *gyrA, gyrB, parC, qnrA*, and *qnrS* which interfere the bacterial DNA synthesis. Since the 1990s, reports on the occurrence of fluoroquinolone-resistant *E. coli* from humans as well as animal have increased significantly [6-8]. Fluoroquinolone-resistant *E. coli* isolates have become a major problem in infection control and treatment worldwide.

In Bangladesh, few previous studies were carried out to investigate the occurrence and characterization of fluoroquinolone- and quinolone-resistant *E. coli* in human [7,8]. Quinolone-resistant *E. coli* has also been detected in healthy cattle and other animals from many other countries [9,10]. Recently, in a study in Bangladesh, quinolone-resistant *E. coli* was detected from apparently healthy cattle [11]. As fluoroquinolones are of major therapeutic importance in human medicine, quinolone-resistant *E. coli* is of great public health concern. Bangladesh is one of the most densely populated countries of the world. The livestock and poultry population, here, are also noticeable. Due to the close contact of human with poultry, there is a great chance for the transmission of antibiotic-resistant *E. coli* from poultry to human.

To the best of our knowledge, no work has yet been reported in Bangladesh on fluoroquinolone resistant in *E. coli* in healthy broiler chickens targeting the detection of *qnrA* and *qnrS* genes by polymerase chain reaction (PCR). The present study was carried out to determine the occurrence of fluoroquinolone-resistant *E. coli* in healthy broiler in Mymensingh, Bangladesh. In addition, the genes responsible for such resistance were also identified by PCR-based approach.

## Materials and Methods

### Ethical approval

No ethical approval was required although all applicable international, national, and institutional guidelines for the care and use of animals were followed during sample collection.

### Collection of samples

A total of 65 cloacal swab samples were collected randomly from apparently healthy broiler chickens sold at Bangladesh Agricultural University (BAU), Mymensingh, and surroundingarea. Among these 65 samples, 23 originated from BAU poultry farm, 5 from Kawatkhali market, 15 from Sesmore market, and 22 from KR market, BAU. Samples were collected maintaining sterile condition and categorized into two age groups: 0-14 days and 15-35 days depending on the age of the birds.

### Isolation and identification of *E. coli*

Isolation and identification of *E. coli* from the collected cloacal swab were done based on morphology, staining, cultural, and biochemical characteristics [12]. Confirmation of the *E. coli* isolates was done by PCR. DNA for the PCR was extracted from pure culture by boiling method. In brief, 100 µl of deionized water was taken into an Eppendorf tube. A pure bacterial colony from overnight culture on 37°C of Eosin Methylene Blue Agar was gently mixed with deionized water. The tube was then transferred into boiling water and boiled for 10 min, then immediately transferred to ice for cold shock for about 10 min, and finally centrifuged at 10,000 rpm for 10 min. Supernatant from each tube was collected and used as PCR DNA template. The extracted DNA was stored at −20°C until use. PCR for *E. coli* 16S rRNA gene was performed using primers ECO-1 and ECO-2 (Table-1) according to the standard protocol [13,14].

**Table-1:** Primers for the detection of *E. coli* 16S rRNA gene, *qnrS* and *qnrS* genes.

Primer name	Target gene	Primer sequence	Amplicon size (bp)	References
ECO-1	16S rRNA gene	5’- GACCTCGGTTTAGTTCACAGA-3’	585	[13]
ECO-2		5’- CACACGCTGACGCTGACCA-3’		
qnrAF	*qnrA*	5’- ATTTCTCACGCCAGGATTTG-3’	516	[14]
qnrAR		5’- GATCGGCAAAGGTTAGGTCA-3’		
qnrSF	*qnrS*	5’- ACGACATTCGTCAACTGCAA-3’	417	[14]
qnrSR		5’- TAAATTGGCACCCTGTAGGC-3’		

E. coli=Escherichia coli

### Antibiotic sensitivity test

The isolated *E. coli* isolates were subjected to antibiotic sensitivity test against five commonly used fluoroquinolone, i.e., gatifloxacin (5 µg), levofloxacin (5 µg), moxifloxacin (5 µg), ofloxacin (5 µg), and pefloxacin (5 µg), following disc diffusion method, as described by Bauer *et al*. [15]. All the tests were performed on Mueller-Hinton media with a concentration of bacteria equivalent to 0.5 McFarland standards. Results of the antibiotic sensitivity tests were recorded as sensitive, intermediately sensitive, or resistant, and the zone of growth inhibition was compared with the zone size interpretative tables provided by the Clinical and Laboratory Standards Institute [16].

### Molecular detection of *qnrA* and *qnrS* genes

Molecular detection of *E. coli* carrying fluoroquinolone-resistant genes (*qnrA* and *qnrS*) was performed by PCR [14]. For this purpose, DNA was extracted from *E. coli* that was found resistant to quinolone phenotypically by the boiling method as described above. The primers used for the detection of *qnrA* and *qnrS* are presented in Table-1 [13,14].

## Results

### Isolation of *E. coli*

Among the 65 cloacal samples analyzed, 54 (83.08%) were found to be positive for *E. coli* (Table-2). On age basis, the highest prevalence of *E. coli* was found in 15-35-day-old healthy broiler chickens (88.07%), while the prevalence was 73.91% in 0-14-day-old birds.

**Table-2:** Prevalence of *E. coli* in the cloacal swab of apparently healthy broiler chickens.

Age groups (days)	Number of samples tested (n)	Number of samples found positive for *E. coli* (n)	Prevalence (%)
0-14	23	17	73.91
15-35	42	37	88.07
Total	65	54	83.08

E. coli=Escherichia coli

### Phenotypic detection of fluoroquinolone-resistant *E. coli*

**Figure-1:**
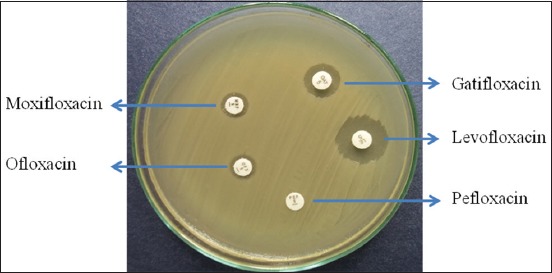
Antibiogram profile of a multidrug-resistant *Escherichia coli* isolated from the cloacal swab of an apparently healthy broiler chicken showing resistance to moxifloxacin, ofloxacin and pefloxacin and sensitive to gatifloxacin and levofloxacin.

All the isolates of *E. coli* (n=54) were screened for antibiogram profile against the five fluoroquinolone group of antibiotics. Among these, 18 (33.33%) isolates were found resistant to at least one fluoroquinolone antibiotic (Figure-1). To be sure that these are *E. coli*, PCR was done, and all these 18 isolates were found positive for *E. coli* 16S rRNA genes (data were not shown).

From Table-3, it is evident that, in general, fluoroquinolone resistance was higher in *E. coil* isolated from the older birds, i.e., 15-35 days old as compared to younger birds of 0-14 days old. The overall antibiogram profile of the isolated *E. coli* against vari ous groups of quinolone groups of antibiotics is presented in Table-4. From the antibiogram profile, it was observed that the highest resistance was found against pefloxacin (61.11%), while the levofloxacin showed less percentage of resistance (22.22%).

**Table-3:** Antibiogram profile of the *E. coli* isolated from the cloacal swab of apparently healthy broiler chickens against the quinolone antibiotics.

Age groups (days)	Number of *E. coli* tested (n)	Sensitivity pattern	Sensitivity pattern on number of *E. coli* against fluoroquinolone antibiotics				

			Gati	Levo	Moxi	Peflo	Oflo
0-14	06	Sensitive	1	3	2	2	3
		Intermediate	2	2	1	0	1
		Resistant	3	1	3	4	2
15-35	12	Sensitive	5	6	2	1	2
		Intermediate	3	3	3	4	2
		Resistant	4	3	7	7	8

Gati=Gatifloxacin, Levo=Levofloxacin, Moxi=Moxifloxacin, Peflo=Pefloxacin, Oflo=Ofloxacin, *E. coli=Escherichia coli*

### Molecular detection of *qnrA* and *qnrS* genes

**Figure-2:**
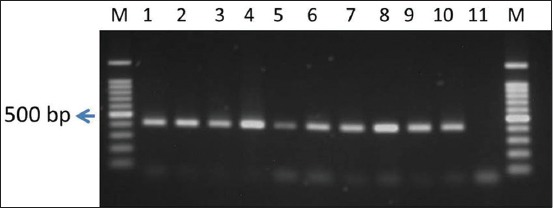
Polymerase chain reaction amplification of *qnrS* gene of *Escherichia coli* isolated from apparently healthy broiler chicken. M - 100 bp size DNA marker; lanes 1-9 - Representative *E. coli*, lane 10 - Positive control and lane 11 - Negative control

Eighteen *E. coli* that showed phenotypically resistant to one or more quinolone group of antibiotics were PCR screened for the detection of *qnrA* and *qnrS* genes. None of the isolates were found positive for *qnrA* (prevalence 0%). On the other hand, among 18 isolates, 13 (prevalence 72.22%) were found positive (Table-5) for *qnrS* gene amplifying 417-bp amplicon (Figure-2).

**Table-4:** The overall prevalence of fluoroquinolone-resistant *E. coli*.

Name of fluoroquinolone antibiotics	Number of isolates found resistant		

	Age of birds		Total (%)

	0-14 days	15-35 days	
Gatifloxacin	3	4	7 (38.89)
Levofloxacin	1	3	4 (22.22)
Moxifloxacin	3	7	10 (50.00)
Pefloxacin	4	7	11 (61.11)
Ofloxacin	2	8	10 (55.00)

E. coli=Escherichia coli

**Table-5:** Prevalence of *qnrS* genes in the fluoroquinolone-resistant *E. coli* as detected by PCR.

Age group (days)	Number of *E. coli* tested by PCR	Number of *E. coli* found positive for *qnrA* (prevalence)	Number of *E. coli* found positive for *qnrS* (prevalence)
0-14	6	0 (0%)	4 (66.66%)
15-35	12	0 (0%)	9 (81.82%)
Total	18	0 (0%)	13 (72.22%)

*E. coli=Escherichia coli*, PCR=Polymerase chain reaction

On age bases, the prevalence of *qnrS* was found higher in older birds, i.e., 15-35-day-old birds compared to younger 0-14-day-old birds.

## Discussion

Antibiotic-resistant bacteria are of great public health concern since they possess a significant threat to human and animal health. *E. coli* is ubiquitous in nature and found commonly as intestinal flora of animal and birds. Many of these *E. coli* are pathogenic and zoonotic in nature. Once they become antibiotic resistant, it is very difficult to treat the disease caused by them. Increasing bacterial resistance to quinolone antibiotics is apparent in humans, livestock, and poultry. The potential source of these resistant bacteria for human is livestock and poultry or their products entering the human food chain.

Fluoroquinolones are broad-spectrum antimicrobial agents, used extensively in poultry [17]. Fluoroquinolone-resistant genes such as *gyrA, gyrB*, and *parC* are located in chromosome, while *gnr* genes are located in plasmid. In this study, we focused on this plasmid-mediated quinolone resistance since these *qnr* genes containing plasmid have potentiality for transfer of resistance to other bacteria horizontally [18]. In addition, these plasmid harboring *qnr* genes may also encode extended-spectrum beta-lactamases such as CTX-M, SHV, and TEM type [19].

In this study, about 83.03% apparently healthy broiler chickens were found to be positive for the presence of *E. coli* in their cloacal swabs (Table-2). Since *E. coli* is a normal inhabitant of birds’ lower gastrointestinal tract, it was not unexpected to find them in higher percentage in the cloacal samples. There are previous reports on 60–82% prevalence of *E. coli* in apparently healthy chickens in Mymensingh, Bangladesh [20,21]. These observed variations in the prevalence of *E. coli* in healthy chickens could be due to variations in the hygienic status of farms, farm biosecurity, ventilation system, sample size, and location of the study area. In this study, we observed a higher prevalence of *E. coli* in older birds as compared to younger bird as supported by earlier reports [22]. This observed a higher prevalence of *E. coli* in older birds might be due to their longer duration of exposure to unhygienic environment, unhealthy management, poor feeding, and watering as compared to younger birds.

Fluoroquinolone antibiotics are widely used in poultry to treat diseases caused by *E. coli*. Antibiogram profile observed in this study revealed 33.33% *E. coli* as resistant to one or more fluoroquinolone group of antibiotics phenotypically. This resistance was higher in older birds as compared to younger birds. It is alarming that, here, we found some of the isolates as multidrug-resistant (MDR) showing resistant against two or three fluoroquinolone group of antibiotic (Figure-1). In a report from Czech Republic, the resistance of *E. coli* of poultry isolates to quinolones ranged from 53% to 73%. Among these resistant phenotypes, resistant genes were detected in 58% of the tested strains [4]. Among the quinolone-resistant genes, *qnrA* and *qnrS* are important. In this study, among the resistant phenotype, about 72.22% isolates were found positive for *qnrS* gene, while none was found positive for *qnrA*. Earlier in Sweden, an increased quinolone-resistant *E. coli* was noted in broiler population, despite the lack of a known selective pressure [23]. They also detected quinolone-resistant *qnrS* gene in *E. coli* strain isolated from broiler.

Due to their high efficacy, quinolones are widely prescribed antibiotic classes for the treatment of human infections caused by bacteria and have been considered critically important for human health by the WHO [24]. The presence of high level of fluoroquinolone-resistant *E. coli* in healthy broiler chicken as detected in this study is, therefore, very important from the public health point of view. Since human can get these resistant isolates from poultry, the use of fluoroquinolones in poultry has to be monitored very carefully.

## Conclusion

The present study reveals the occurrence of fluoroquinolone-resistant *E*. *coli* in healthy broiler. Some of the isolates are MDR. This is the first report of the detection of *qnrS* gene in *E*. *coli* isolated from healthy broiler chickens in Bangladesh which are resistant to fluoroquinolone group of antibiotics. From poultry, these resistant bacteria can be transfered to human through the food chain. It is now time to be careful in the use of fluoroquinolone and quinolone group antibiotics in poultry in Bangladesh.

## Authors’ Contributions

MTR and KHMNHN: Designed the study. SM did laboratory work and assisted by MTR and KHMNHN. MTR and SM prepared the manuscript and analyzed the data with the help of KHMNHN. All authors read and approved the final manuscript.
